# Prevalence of hand osteoarthritis and knee osteoarthritis in Kashin-Beck disease endemic areas and non Kashin-Beck disease endemic areas: A status survey

**DOI:** 10.1371/journal.pone.0190505

**Published:** 2018-01-10

**Authors:** Wei Lian, Hui Liu, QuanQuan Song, Yun Qi Liu, Li Yan Sun, Qing Deng, Shao Ping Wang, Yan Hong Cao, Xue Ying Zhang, Yuan Yuan Jiang, Hong Yan Lv, Li Bin Duan, Jun Yu

**Affiliations:** 1 Institute for Kashin-Beck Disease Control and Prevention, Chinese Center for Disease Control and Prevention, Harbin Medical University, Harbin, China; 2 Key Laboratory of Etiology and Epidemiology, National Health and Family Planning Commission, Harbin, China; 3 Jilin Institute of Endemic Disease Prevention second, Jilin, China; University of Umeå, SWEDEN

## Abstract

Osteoarthritis (OA) is a considerable health problem worldwide, and the prevalence of OA varies in different regions. In this study, the prevalence of OA in Kashin-Beck disease (KBD) and non-KBD endemic areas was examined, respectively. According to monitoring data, 4 types of regions (including none, mild, moderate and high KBD endemic areas) in Heilongjiang and Jilin provinces were selected. All local residents were eligible for inclusion criteria have undergone X-ray images of hands and anteroposterior image of knees. A total of 1673 cases were collected, 1446 cases were analyzed after removing the KBD patients (227). The overall hand OA and knee OA detection rates were 33.3% (481/1446) and 56.6% (818/1446), respectively. After being standardized by age, the detection rate of hand OA in the KBD endemic areas was significantly higher than that in the non-endemic endemic areas. Differently, there was no significant difference in the detection rates of knee OA between the KBD endemic areas and the non-endemic area. The correlation coefficient between the severity of OA and the severity of knee OA was 0.358 and 0.197 in the KBD and non-KBD endemic areas, respectively. Where the KBD historical prevalence level was higher, the severity of the residents’ hand OA was more serious. The detection rates of hand OA and knee OA increased with age. The detection rate of knee OA increased with the increase in body mass index. The prevalence of hand OA was closely related to the pathogenic factors of Kashin-Beck disease, and the prevalence of knee OA had no significant correlation with KBD pathogenic factors.

## Introduction

Osteoarthritis (OA) is a low-grade inflammatory disease of synovial joints and the most common form of arthritis [1]. It is a leading cause of chronic pain and physical disability in older individuals [2]. OA is one of the most costly and disabling forms of joint disease, being far more common than rheumatoid arthritis (RA) and other forms of joint disease [3]. It is characterized by progressive deterioration and loss of articular cartilage [4] with concomitant structural and functional changes in the entire joint, including the synovium, meniscus (in the knee), periarticular ligaments, and subchondral bone [5].

Kashin-Beck disease (KBD) is a regional, symmetrical, disease of multiple deformed bones and joints [6], and it was named such by the international medical community [7]. The disease can occur in all parts of the body and is known for the formation of multi-joint hyperplasia bone changes [8]. In China, KBD is mainly distributed in narrow areas stretching from the northeast to the southwest. The disease can also be found in Siberia and a few areas in North Korea. Most of the endemic areas are located in the cold and arid regions of warm and humid areas [9].

Previous studies have shown that the prevalence and severity levels of hand OA differed between the areas where the staple food was rice or flour [10]. The geographical distribution of the results of Yang Jianbo et al regarding the X-ray detection of adult OA of the hand bone also showed that the prevalence was higher in the north, with the most serious cases found in the KBD areas and its surrounding areas; the south showed less hand OA, with the lowest prevalence in the Jiangsu and Zhejiang area [11]. In the past, the people in KBD area usually ate flour as their staple food [12].

Currently, little research exists on the prevalence of OA in the KBD endemic areas. Therefore, in this study, we want to know whether the prevalence and severity levels of OA in the KBD areas are higher than in the non-endemic areas. Our study added more epidemiological data of osteoarthritis by describing and analyzing the prevalence and severity levels of OA in the KBD areas and the non-endemic areas.

## Materials and methods

### Investigation site

Using a typical survey according to monitoring data, we selected 4 types of regions in Heilongjiang and Jilin provinces, including a mild, a moderate, and a severe prevalence KBD area as well as a non-KBD endemic area. From each region, 1–5 villages were selected, and all of the local residents older than 39 years old in each selected village were examined.

Shuangzhi village and Xinglong village (Yanshou county of Heilongjiang province) in KBD severe area; Dongxia village and Zhoujia village (Songyuan city of Jilin province), Hanxia village and Youhao village (Jiaohe city of Jilin province), and Changyou village (Tieli city of Heilongjiang province) in KBD moderate area; Xinping village and Xichuan village (A’cheng city of Heilongjiang province) in KBD mild area; Heigang village (Qiqihaer city of Heilongjiang province), Sanjing village (Songyuan city of Jilin province), and Fuqiang village (Jiaohe city of Jilin province) in non-endemic area were selected as our investigation sites, respectively.

### Study subject and investigation contents

At least 50 people underwent clinical examination in the selected village, and their age is over 40 years old. The research contents included height, weight, life habits, medical history, hand’ and knee’ X-ray images, etc. S1 Table.

### Detection equipment and methods

#### Detection equipment

High frequency portable digital medical diagnostic X-ray image DR system (Beijing Longan Imaging Technology limited company).

(1) X-ray generator

Exposure time: 0.01s-4.0s Focus size: 2.3mm

mAs adjustment range: 0.5–160 mAs KV adjustment range 40–90 KV

(2) Flat panel detectors

Pixels: 2208×2688 Pixels Image size: 356×427mm (14×17 in)

(3) Portable video workstation

Computer model: Lenovo computer Y450

#### Detection method

Detection site: metacarpophalangeal joint, knee joint

(1) Shoot the hands of the image: palm down, fingers close together, straight, flat on the DR system image receiving screen, wrist and hand to be kept in a straight line.

(2) Shoot the knees of the image: the legs straight, knee flat on the DR system image receiving screen.

#### Instrument parameter setting

The parameters of the all-digital multi-function radiography system (DR) were:

(1) Shoot the hands of the image

Exposure conditions: 50KV, 2.86 mAs Projection distance: 100 cm

(2) Shoot the knees of the image

Exposure conditions: 60KV, 4.20 mAs Projection distance: 100 cm

### Quality control

In order to ensure the quality of the images, all abnormal X-ray images were examined by the experts; if the results were inconsistent with the image again, the findings were considered diagnostic. The results were combined and underwent statistical analysis.

### Judgement standard

#### Investigate village selection criteria

KBD investigation of the village selection principle: ① history of being a KBD endemic area; ② KBD monitoring data complete; ③ X-ray positive detection rate of children <3%; ④ the population of residents over age 40 can not be less than 100 people.

Non-KBD investigation of the village selection criteria: ① no history of KBD; ② the population of residents over age 40 can not be less than 100 people; ③ similar eating habits and economic level to KBD villages.

#### KBD endemic area identification and division criteria

Mild endemic area: prevalence of the local residents of clinical I grade and above or X-ray detection rate of ≤ 10% in 7-12-year-old children.

Moderate endemic area: prevalence of the local residents of clinical I degrees and above or X-ray detection rate > 10% and ≤ 20% in 7-12- year-old children.

Severe endemic area: prevalence of the local residents of clinical I degrees and above or X-ray detection rate> 20% in 7-12-year-old children.

### KBD diagnostic criteria

Kashin-Beck disease was diagnosed in accordance with the "Kashin-Beck disease diagnosis" standard (WS / T 207–2010) for the diagnosis of Kashin-Beck disease. Osteoarthritis was diagnosed in accordance with the American College of Rheumatology 1995 diagnostic criteria.

KBD is an osteoarthropathy with joint injury of limbs. The X-ray image of hand can reflect the degree of pathological lesions and the damage of the joints in the whole body, and there is no difference between the change of large joints, such as the elbow in KBD and that in OA. Therefore, we can regard the X-ray image of hand as the basis of diagnosis and differential diagnosis. KBD patients with hand X-ray images often manifested as multiple, symmetrical finger joint thickening or short finger deformity, severe appearance of short stature. KBD patients with the characteristics of the hand X-ray images for basal broadening or spindle-shaped of the proximal end of phalanx, horn mouth shape change of proximal end of phalanx, collapse repair phase of phalanx, which is not available in the hand X-ray of patients with osteoarthritis.

### Image classification standards

#### Palm finger joint imaging classification criteria

**Fig 1:**
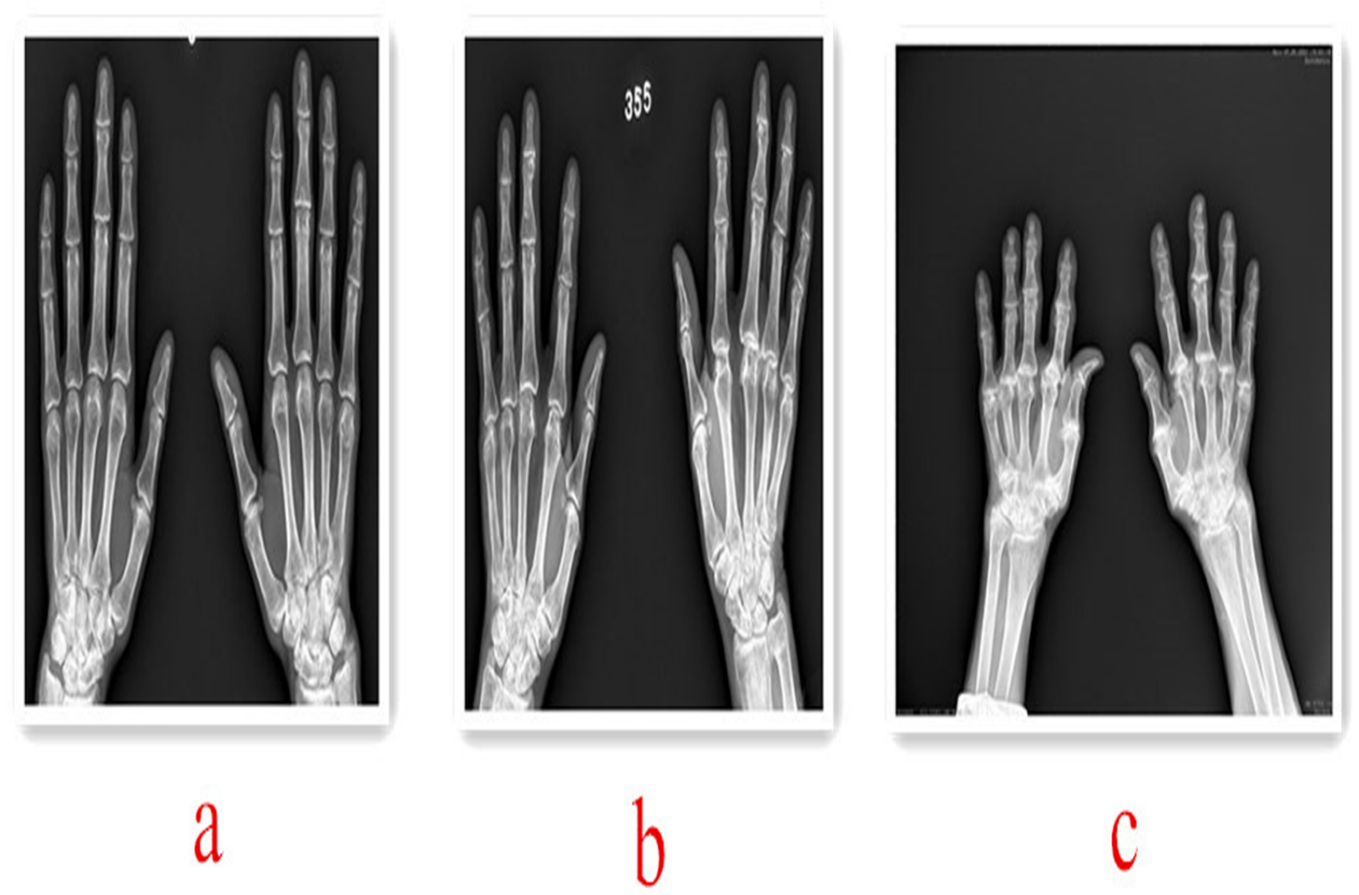
Different degrees of hand OA imaging. **a) Mild hand OA.** Right middle finger index distal articular surface is not the whole, middle finger proximal and small finger base proximal cystic change, middle finger base distal ulnar hyperplasia and periosteal reaction. b) **Moderate hand OA.** Middle finger proximal ulnar side of the lateral hyperplasia, metacarpal head sickle-like, right palm of the head articular surface hardening, base section of the phalanx to the radial side skew. c) **Severe hand OA.** Base widened, ulnar side edge hyperplasia, articular surface hardening. Metacarpal swelling of the metacarpal bone with sickle, articular surface hardening, metacarpophalangeal joint narrowing, left hand 2,3, right hand 2,3,4 nodules to the radial side skew. Carpal edge hardening deformation.

**Mild**: ① joint stenosis; ② distal phalanx, proximal margin of the formation of the angle or small spur or small low density proliferation; ③subchondral bone sclerosis; ④subchondral cystic change; ⑤ cortical collapse; ⑥ carpal crowded, disorder. (Fig 1a)

**Moderate**: ① distal phalanx, proximal margin of the formation of bone crest, bone spine; ② metacarpal head sickle shadow; ③ carpal edge hardening, not the whole or hyperplasia. (Fig 1b)

**Severe**: ① distal brachial flexion and subluxation, metacarpophalangeal subluxation; ② metacarpal hypertrophy with sickle-like; ③ carpal deformation. (Fig 1c)

### Classification of knee imaging

**Fig 2:**
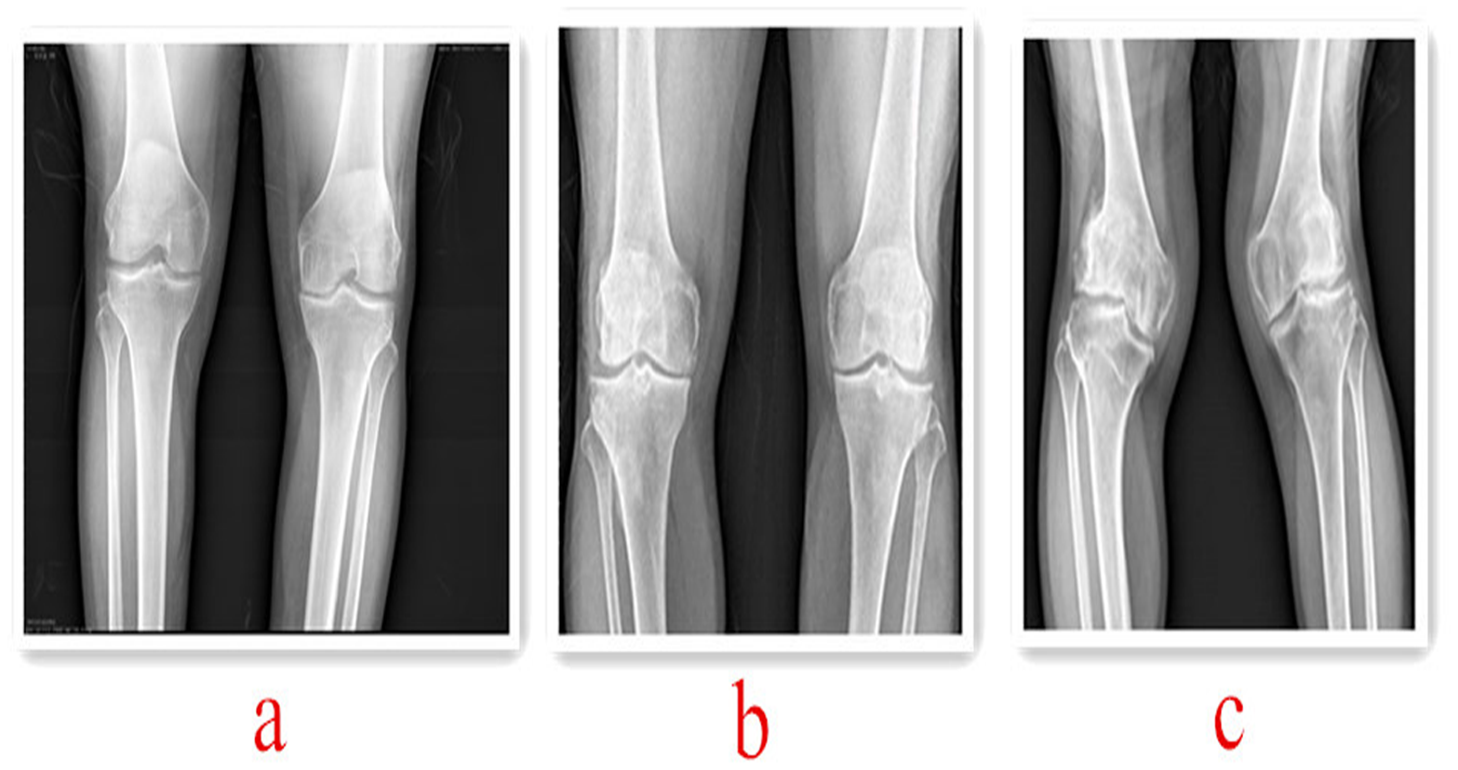
Different degrees of knee OA imaging. **a) Mild knee OA.** Intercondylar prolapse ossification. b) **Moderate knee OA.** Intercondylar prolapse ossification, proximal tibial lip-like hyperplasia, uneven gap. c) **Severe knee OA.** Distal femur, proximal tibial enlargement, uneven knee joint, articular surface irregularities, intercondylar prolapse ossification.

**Mild**: intercondylar uplift slightly ossification, no clinical symptoms or mild clinical symptoms. (Fig 2a)

**Moderate**: intercondylar interria obvious ossification, joint space narrowing or uneven width, distal femur or proximal tibial lip-like hyperplasia, clinical symptoms were obvious. (Fig 2b)

**Severe**: joint swelling and deformation, intercondylar intervertebral ossification prominent, joint space is cloudy, with narrow stenosis or uneven width, distal femur or proximal tibial lip-like hyperplasia, difficult to walk. (Fig 2c)

### Statistical methods

SPSS 18.0 statistical software was used for statistical analysis. The detection rate of arthritis was compared using a chi-square test. Furthermore, the difference between the two groups was analyzed using the card separation method; the mean number was chosen to be t test, and the test level was chosen to be α = 0.05.

The study was performed in accordance with the Declaration of Helsinki and approved by the Human Ethics Committee of Endemic Disease Center of Harbin Medical University, PR of China. Written informed consent was also obtained from the subjects S1 File.

## Results

Table 1 provides the overall inspection results for each region. In this study, 1673 effective samples were collected from 12 villages in 2 provinces. A total of 227 KBD patients were detected, and the detection rate was 13.6%. Including KBD patients, hand OA was detected in 708 people, with a detection rate of 42.3%. Knee OA was detected in 1045 people, with a detection rate of 62.5%. Excluding KBD patients, the remaining sample included 1446 people; 481 of these patients were found to have hand OA, a detection rate of 33.3%. The detection rate in the KBD endemic areas was significantly higher than in the non-KBD endemic area (χ^2^ = 79.910, P < 0.001). There were 818 knee OA patients with a detection rate of 56.6%; the detection rate in the KBD endemic areas was significantly higher than in the non-KBD endemic area (χ^2^ = 215.789, P < 0.001).

**Table 1:** Overall inspection results for each region.

Survey area	Name of the village	N	Hand OA		Knee OA		KBD	
			n	Detection rate (%)	n	Detection rate (%)	n	Detection rate (%)
**Severe area**	Shuangzhi	64	43	67.2	46	71.9	25	39.1
	Xinglong	51	34	66.7	32	62.7	20	39.2
	Changyou	128	73	57.0	86	67.2	46	35.9
**Moderate area**	Dongxia	125	71	56.8	95	76.0	18	14.4
	Zhoujia	89	60	67.4	73	82.0	27	30.3
	Hanxia	183	79	43.2	115	62.8	16	8.7
	Youhao	223	96	43.0	150	67.3	32	14.3
**Mild area**	Xinping	87	43	49.4	45	51.7	21	24.1
	Xinchuan	85	45	52.9	38	44.7	22	25.9
**Non-endemic area**	Heigang	341	74	21.7	203	59.5	0	0
	Sanjing	114	37	32.5	66	57.9	0	0
	Fuqiang	183	53	29.0	96	52.5	0	0

Table 2 provides the basic characteristics of the subjects after removal of KBD.

**Table 2:** Basic characteristics of the subjects after removal of KBD.

Region	N	Age (years)	Sex (male/female)	BMI (kg/m^2^)
**Non-endemic area**	638	57.61±9.85	223/415	23.92±3.59
**KBD area**	808	57.19±9.34	277/531	24.85±3.56

### Gender differences in hand OA and knee OA

**Fig 3:**
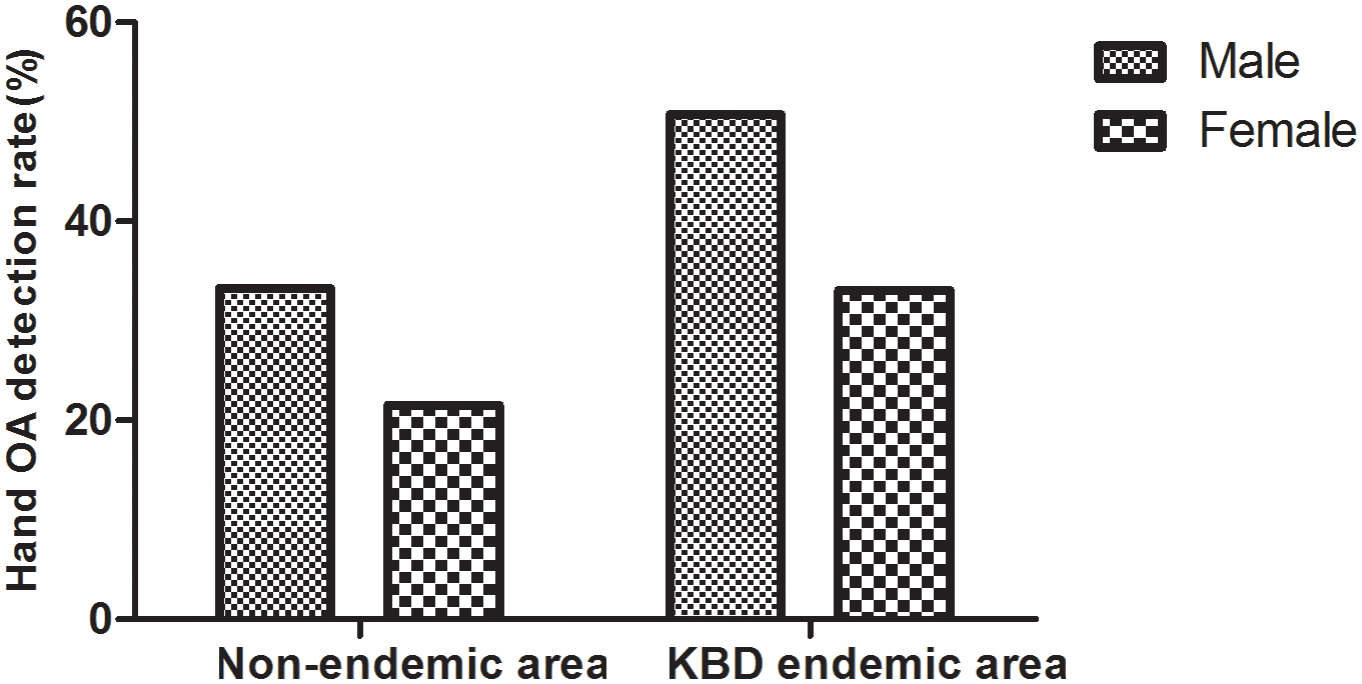
The detection rate of hand OA in the different genders.

In the KBD endemic area, the male detection rate of hand OA was 50.7% (143/282), and the female detection rate was 33.1% (174/526). In the non-endemic area, the male detection rate was 33.3% (75/225), and the female detection rate was 21.5% (89/413) (Fig 3). There were no statistically significant differences in gender composition between the two groups (χ^2^ = 0.021, P = 0.885). Regardless of the status as endemic area or non-endemic area, the detection rate of hand OA in male patients was higher than in females (χ^2^ = 23.932, P < 0.001; χ^2^ = 10.590, P = 0.001).

**Fig 4:**
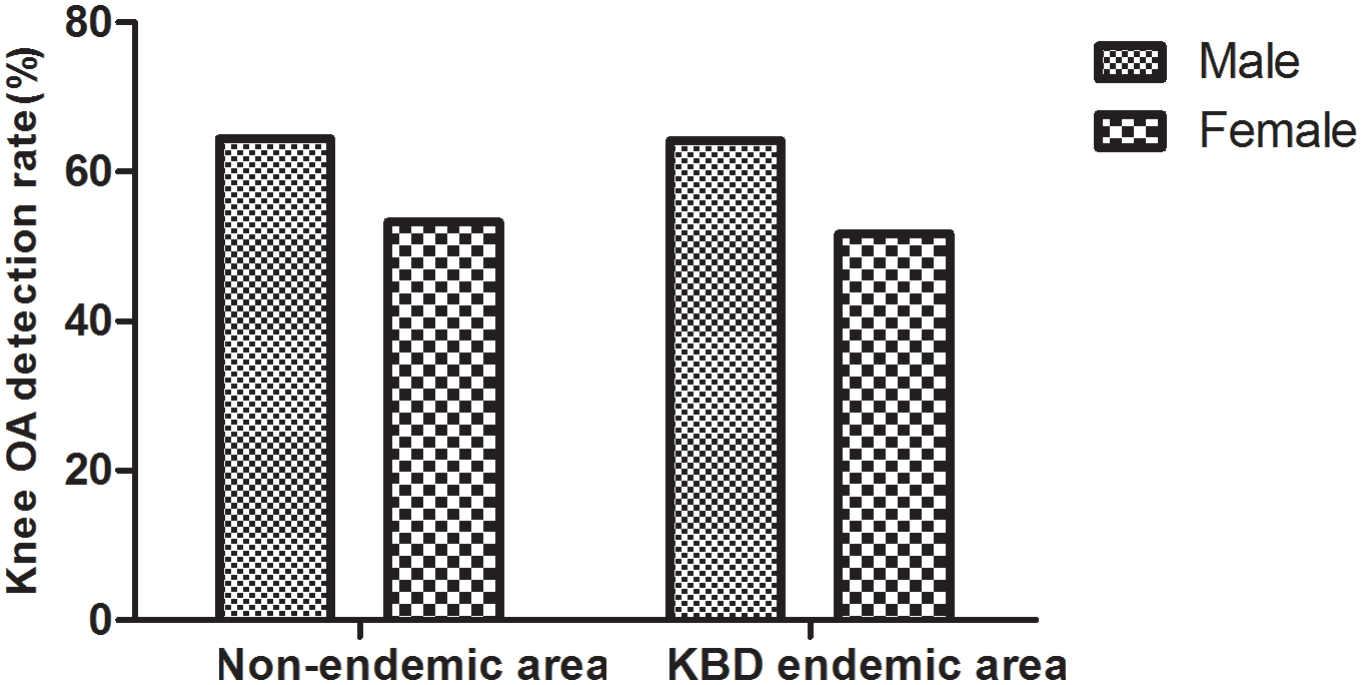
The detection rate of knee OA in the different genders.

In the KBD endemic area, the male detection rate of knee OA was 64.2% (181/282), and the female detection rate was 51.7% (272/526). In the non-endemic area, the male detection rate of knee OA was 64.4% (145/225), and the female detection rate was 53.3% (220/413) (Fig 4). Regardless of the status as an endemic area or non-endemic area, the detection rate of knee OA in male patients was higher than in females (χ^2^ = 11.595, P = 0.001; χ^2^ = 7.431, P = 0.006).

### Age difference in hand OA and knee OA

**Fig 5:**
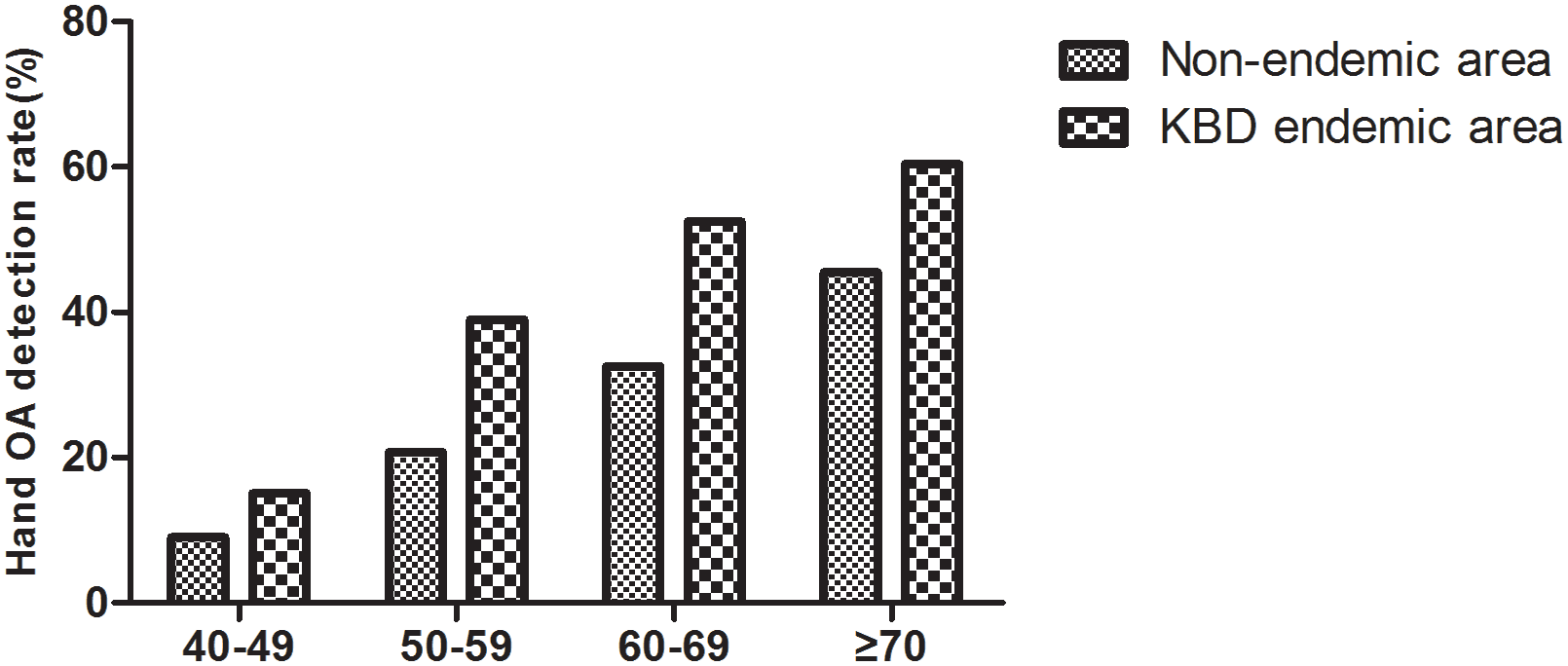
The detection of hand OA in different age groups in the two groups.

There was a significant difference in the detection rate of hand OA between the KBD endemic area and the non-endemic area (χ^2^ = 89.790, P < 0.001; χ^2^ = 48.888, P < 0.001), and the detection rate of OA increased with age (Fig 5). To compare the detection rate of hand OA at the same age between the KBD endemic area and the non-endemic area, the rate of hand OA detection in the 40-49-year-old age group was not statistically different (χ^2^ = 2.602, P = 0.107); however, the hand OA detection rates of three age groups were higher in the endemic area than in the non-endemic area (χ^2^ = 17.867, P < 0.001; χ^2^ = 18.869, P < 0.001; χ^2^ = 3.926, P = 0.048).

**Fig 6:**
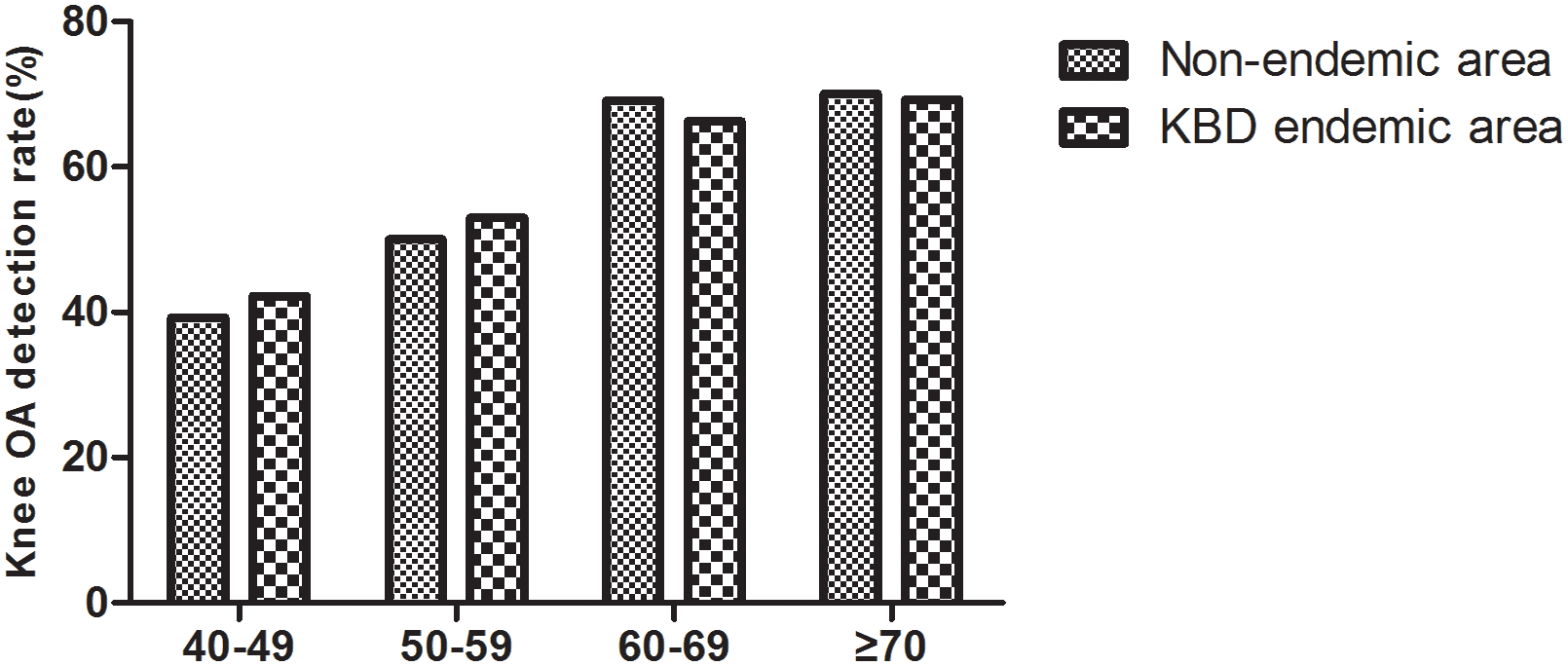
The detection of knee OA in different age groups in the two groups.

There was a significant difference in the detection rate of knee OA between the KBD endemic area and the non-endemic area (χ^2^ = 35.248, P < 0.001; χ^2^ = 38.908, P < 0.001), and the detection rate of OA increased with age (Fig 6). When comparing the detection rate of knee OA at the same age between the KBD endemic area and the non-endemic area, the detection rate of the endemic area was slightly higher than in the non-endemic area, but there was no significant difference in the detection rate of knee OA (χ^2^ = 0.264, P = 0.608; χ^2^ = 0.416, P = 0.519; χ^2^ = 0.452, P = 0.501; χ^2^ = 0.014, P = 0.904).

### Relationship between knee OA and body mass index

By statistical test, the body mass index of knee OA patients was higher than in the non-knee OA group (t = -4.900, P < 0.001) (Table 3). With an increase in BMI, the detection rate of knee OA gradually increased (Table 4).

**Table 3:** Body mass index in patients with OA and Non-OA.

	N	Mean	Standard deviation
**Non-knee OA**	628	23.92	3.59
**Knee OA**	818	24.85	3.56

**Table 4:** Relationship between BMI and number of case of knee OA.

BMI	N	Knee OA	Rate(%)
**I(BMI<18.5)**	53	24	45.2
**II(18.5≤BMI<24)**	630	314	49.8
**III(24≤BMI<28)**	535	327	61.1
**IV(BMI≥28)**	228	153	67.1

### Age of the standardized population hand OA and knee OA detection rates

The total number of people surveyed was the standard population. From this number, the number of expected detections and the standardized hand OA detection rate were calculated (Table 5). The detection rate of hand OA in the KBD endemic area was higher than in the non-endemic area, and the difference was statistically significant (χ^2^ = 79.910, P < 0.001).

**Table 5:** Age of the standardized population hand OA detection rate in the KBD area and non-endemic area.

Region	Standardized population		
	N	Standard population	Rate(%)
**Non-endemic area**	360	1446	24.9
**KBD area**	584	1446	40.4

After age was standardized (Table 6), the detection rate of hand OA in the four groups was statistically different (χ^2^ = 656.092, P < 0.001). Hand OA was detected most frequently in the seriously KBD endemic area and was the lowest in the non-endemic area. Furthermore, the chi-square segmentation method was used to compare the two; the detection rate of hand OA in the seriously endemic area was higher than in the moderate endemic area (χ^2^ = 185.856, P < 0.001). The detection rate of the moderate endemic area was higher than in the mild endemic area (χ^2^ = 194.327, P < 0.001). The mild endemic area was higher than the non-endemic area (χ^2^ = 191.186, P < 0.001).

**Table 6:** Detection rate of hand OA in each region.

Region	Standardized population		
	N	Standard population	Rate(%)
**Severe**	670	1446	46.3
**Moderate**	601	1446	41.6
**Mild**	485	1446	33.5
**Non-endemic area**	360	1446	24.9

After age was standardized (Table 7), the detection rate of knee OA in the KBD endemic area and the non-endemic area was no significant difference (χ^2^ = 0.114, P = 0.736).

**Table 7:** Age of the standardized population knee OA detection rate in the KBD area and non-endemic area.

Region	Standardized population		
	N	Standard population	Rate(%)
**KBD area**	821	1446	56.8
**Non-endemic area**	812	1446	56.2

The four regions were standardized according to age; the highest detection rate was in the moderate area, and the lowest detection rate was in the mild endemic area (Table 8). A further chi-square segmentation method was used to compare the two, and the detection rate of knee OA in the moderate area was higher than in the non-endemic area (χ^2^ = 215.789, P < 0.001); the non-endemic area was higher than the severe endemic area (χ^2^ = 183.721, P < 0.001); and the severe endemic area was higher than the mild endemic area (χ^2^ = 276.237, P < 0.001).

**Table 8:** Detection rate of knee OA in each region.

Region	Standardized population		
	N	Standard population	Rate(%)
**Severe**	684	1446	47.3
**Moderate**	919	1446	63.6
**Mild**	415	1446	28.7
**Non-endemic area**	812	1446	56.2

### Age of the standardized endemic areas and non-endemic area hand OA and knee OA severity of the detection of the situation

**Fig 7:**
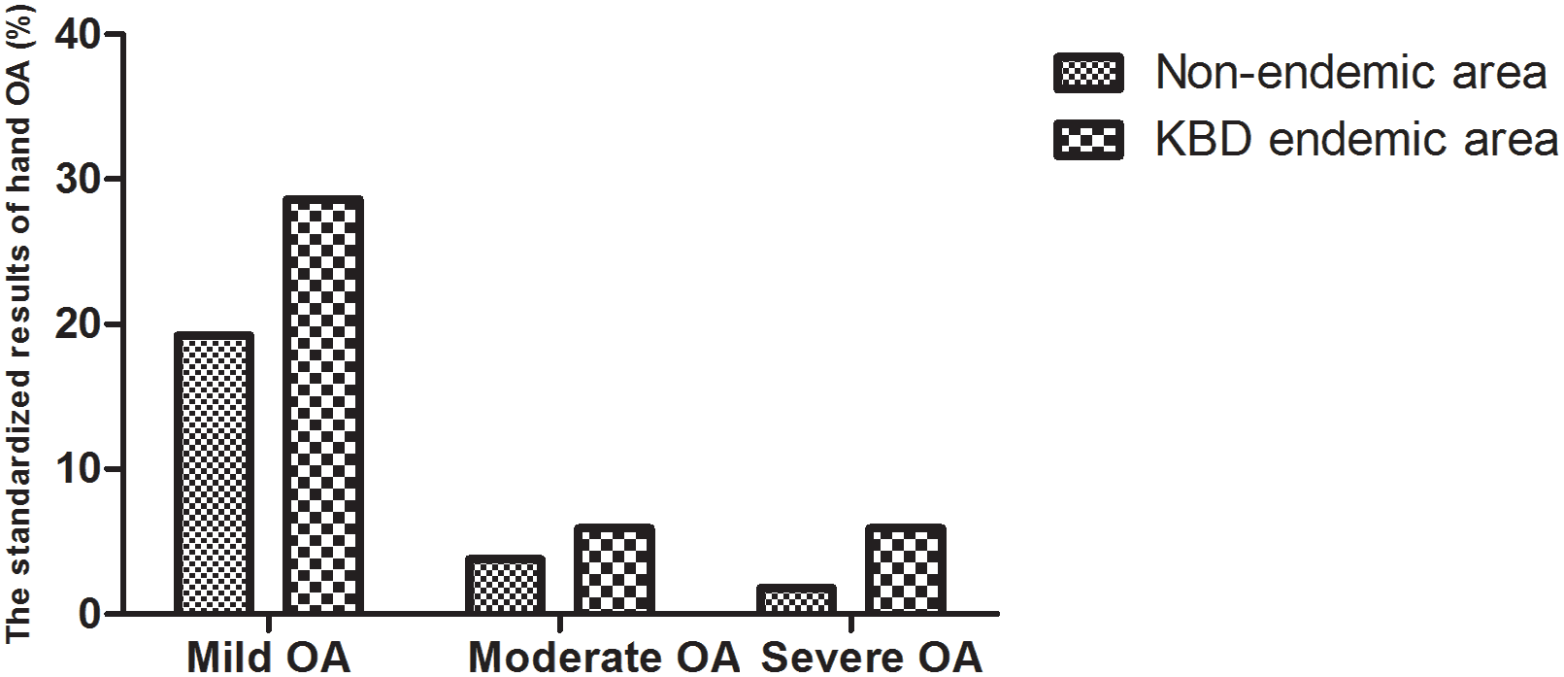
The standardized results of different degrees of hand OA in the non-endemic area and KBD endemic area.

The detection rate of mild, moderate and severe hand OA in the KBD endemic area was 28.6%, 5.9% and 5.9%, respectively. The detection rate of mild, moderate and severe hand OA in the non-KBD endemic area was 19.2%, 3.8% and 1.8%, respectively. The detection rate of mild, moderate and severe hand OA in the two regions was statistically different (χ^2^ = 34.655, P < 0.001; χ^2^ = 7.165, P = 0.007; χ^2^ = 32.612, P < 0.001), and the detection rate of mild, moderate and severe hand OA in the KBD endemic area was significantly higher than in the non-endemic area (Fig 7).

**Fig 8:**
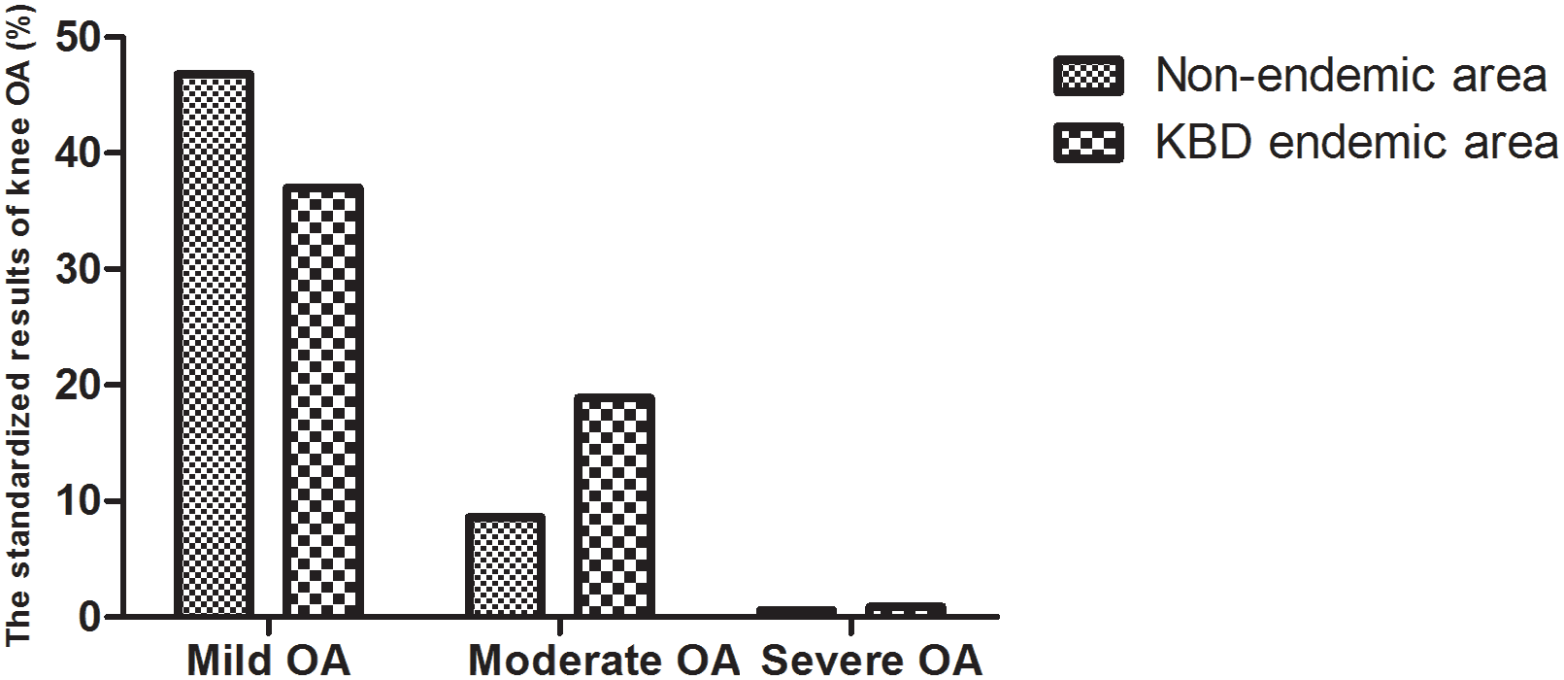
The standardized results of different degrees knee OA in the non-endemic area and KBD endemic area.

The two categories of regions were standardized by age, and the detection rate of mild, moderate and severe knee OA in the KBD endemic area was 37.0%, 18.9% and 0.9%, respectively. The detection rate of mild, moderate and severe knee OA in the non-endemic area was 46.8%, 8.6% and 0.6%, respectively. There were statistically significant differences in the detection rate of knee OA between the two groups (χ^2^ = 28.639, P < 0.001; χ^2^ = 64.820, P = 0.007), and there was no significant difference in the detection rate of severe knee OA (χ^2^ = 0.733, P = 0.392). The detection rate of mild knee OA was significantly lower than in the non-endemic area, and the detection rate of moderate knee OA was significantly higher than in the non-endemic area (Fig 8).

### Correlation between the severity of hand OA and the severity of knee OA

A list of 808 subjects in the wrist and knee X-ray reading results were analyzed, and the statistical analysis showed that the severity of hand OA in the KBD endemic area and the severity of knee OA were statistically different (χ^2^ = 121.131, P < 0.001) (Table 9). There was a linear trend relationship between the severity of hand OA and the severity of knee OA, and the correlation coefficient was **r** = 0.358.

**Table 9:** Correlation between the severity of hand OA and the severity of knee OA in the KBD endemic area.

**Hand joint X-ray grading**	**Knee joint X-ray grading**				**Total**
	**Normal**	**Mild**	**Moderate**	**Severe**	
**Normal**	270	171	47	3	491
**Mild**	74	99	52	1	226
**Moderate**	8	16	20	2	46
**Severe**	3	13	28	1	45
**Total**	355	299	147	7	808

A list of 638 subjects in the wrist and knee X-ray reading results were analyzed. The statistical analysis showed that the severity of hand OA in the non-endemic area and the severity of knee OA were statistically different (χ^2^ = 30.424, P < 0.001) (Table 10). There was a linear trend relationship between the severity of hand OA and the severity of knee OA, and the correlation coefficient was **r** = 0.197.

**Table 10:** Correlation between the severity of hand OA and the severity of knee OA in the non-endemic area.

**Hand joint X-ray grading**	**Knee joint X-ray grading**				**Total**
	**Normal**	**Mild**	**Moderate**	**Severe**	
**Normal**	224	219	29	2	474
**Mild**	39	70	17	1	127
**Moderate**	6	10	8	1	25
**Severe**	4	4	3	1	12
**Total**	273	303	57	5	638

## Discussion

The aim of this study was to investigate the effect of KBD pathogenic factors on the development of OA. The results of this survey showed that the detection rate of OA in the KBD endemic areas was higher than in the non-endemic areas, and the heavier the KBD history of the endemic areas, the higher was the detection rate of OA. The results were consistent with those of Yang Jianbo [11]. The detection rate of mild, moderate and severe hand OA in the endemic areas was significantly higher than in the non-endemic areas (Fig 7), indicating that KBD pathogenic factors have a significant impact on the occurrence and development of hand OA. KBD pathogenic factors can lead directly to KBD in childhood in some people, while KBD changes do not occur in early childhood in others. The elderly with pathogenic factors, in addition to advancing age and articular cartilage function decline are more likely to suffer from OA.

This study also found that the detection rate of knee OA in the KBD areas was slightly higher than that in the non-endemic areas, but there was no significant difference, indicating that the effect of KBD pathogenic factors on the occurrence of knee OA was not obvious. The reason for this finding may be that KBD pathogenic factors are not significantly related to the injury of large joints, and factors such as lifestyle and labor intensity have a greater effect on knee OA. However, this study found that the incidence of mild knee OA was significantly lower than in the non-endemic areas, and the moderate knee OA detection rate was significantly higher than in the non-endemic areas (Fig 8), illustrating that pathogenic factors of KBD can impact the development of knee OA.

The results of this survey showed that the severity of hand OA was linearly correlated with the severity of knee OA in the same area, but the severity of hand OA was not representative of the severity of knee OA. Correlation between the severity of hand OA and the severity of knee OA in the KBD areas were higher than in the non-endemic areas, which may be because the prevalence of knee OA in the non-endemic area was higher than hand OA. In the KBD areas, KBD pathogenic factors on the injury of the hand and bone joints increased the severity of hand OA, although the effect on knee OA was not obvious. Therefore, the severities of hand OA and knee OA in the KBD areas were relatively highly correlated.

Estrogen protects articular cartilage, which can directly inhibit the damage of bone and cartilage from cytokines. Studies have shown that in women after menopause, the level of estrogen and progesterone in the body changes significantly, the balance between the two hormones is altered, and the prevalence of OA in postmenopausal women increases [13]. Rong et al showed that the average prevalence of OA was 11.1% and that the average prevalence in women was 26.8% [14]. The survey by Yu Wei et al also showed that the prevalence of knee OA was higher than men [15]. The results of an epidemiological survey of the elderly in six of China’s cities by Li Ninghua et al showed that the prevalence of OA in women in all regions was higher than in men [16]. However, the results that found that the detection rate of hand and knee OA were higher in women, may have surveyed populations with relatively small numbers of healthy men, considering the male labor load in rural areas.

Domestic and foreign surveys show that age is a major risk factor for OA [17, 18]. The greater the age, the greater is the risk of developing OA. There is a trend toward the development of OA characteristics in younger people, as in the study by Zang Changhai et al on the Taiyuan area, which found that after 40 years of age, the prevalence of OA significantly increased [19]. The OA survey of the rural elderly in Shanghai Fengxian by Wang Yongbin et al showed that with increasing age, OA prevalence increased. At 40–50 years old, the rate was 37.6%; at 50–60 years old, the rate had reached 62.7%; and at 60–70 years old and 70 years of age, the rates were 72.7% and 74.6%, respectively [20]. A survey of the Minhang District in Shanghai by Gu Ming Shi et al found 613 cases of OA in residents 61–81 years of age, translating to a knee OA prevalence of up to 60.5% [21]. The results also showed that the detection rate of hand and knee OA significantly increased with age. And the prevalence was heaviest in people over 60.

Obesity has long been identified as a risk factor for knee OA [22]. In a meta-analysis, those individuals who were obese or overweight had a 2.96 times higher risk of incident knee OA compared with those who were normal weight (95% CI 2.56–3.43) [23]. Assuming the prevalence of obesity in a hypothetical population to be 25%, the population attributable risk percent due to obesity would therefore be 29% (95% CI 24–34%); this rate would be higher where the prevalence of obesity is higher [24]. Furthermore, those individuals who were only overweight (not obese) had over 2 times the chance of developing knee OA compared with their normal weight counterparts [23]. Risk of incident knee OA increases with increasing BMI, regardless of knee alignment [25]. Decreasing BMI by 2 units or more over 10 years (~5 kg) was associated with a 50% lower risk of developing symptomatic knee OA among women [26], findings that were supported by a recent meta-analysis [27]. The duration of exposure to high BMI during adulthood confers risk of incident knee OA, suggesting the importance of weight control throughout life as a means of primary prevention of knee OA [28]. The results of this study also showed that the BMI of the knee OA population was higher than that of the non-knee OA population (t = -4.900, P = 0.000), and with the increase in BMI, the detection rate of knee OA gradually increased (Tables 3 and 4).

Our study has some merits. The knee joint is one of the load-bearing joints that has a significant amount of daily activity, is a common joint affected by OA, and is therefore more commonly researched than hand OA both at home and abroad. To date, research on the prevalence of OA in the KBD areas has been minimal. In this study, we investigated the prevalence of OA in the KBD areas and the non-endemic areas by comparing the prevalence of OA in the KBD areas and the non-endemic areas and the differences in the severity of OA in each region, providing the foundation for research on the etiology of KBD and OA. At the same time, we also explored the relationship between the prevalence of knee OA and the prevalence of hand OA.

Some limitations are present in this study. Because in rural areas, men 40–60 years of age go out to work more, the survey population therefore included a relatively small number of healthy men. In addition, according to historical survey data, there were more male KBD patients than female in the KBD areas.

## Conclusion

The present study found that the residents’ hand OA detection rate in the KBD area is higher than in the non-endemic area. Where the KBD historical prevalence level was higher, the residents’ hand OA was more serious. There was no significant difference in the knee OA detection rates between the KBD endemic area and the non-endemic area. The detection rates of hand OA and knee OA increased with age. The detection rate of knee OA increased with an increase in body mass index. The incidence of hand OA was closely related to the pathogenic factors of KBD and the incidence of knee OA had no significant correlation with KBD pathogenic factors.

## Supporting information

S1 Table Adult questionnaire. (DOCX)

S1 File Informed consent. (DOCX)
