# Supplementary material for: Prevalence of hand osteoarthritis and knee osteoarthritis in Kashin-Beck disease endemic areas and non Kashin-Beck disease endemic areas: A status survey
Source: PLoS One. 2018 Jan 10;13(1):e0190505. doi: 10.1371/journal.pone.0190505 (PMC5761882; doi:10.1371/journal.pone.0190505)
Supplement: S1 Table — (DOCX) [file pone.0190505.s001.docx]

**成人调查表**

| ________________省 | ________________市 | ________________县 | | ________________乡（镇） | __________________村 |
| --- | --- | --- | --- | --- | --- |
| 姓名 | 性别： □男 □女 | 年龄： | | 民族： | 调查编号： |
| 生活习惯 饮酒：□从不 □偶尔 □经常 □每天  吸烟：□是 □否  主要食用粮食：□大米 □玉米 □面粉  在该地区居住年限：____________年 | | | 既往病史：__________________  骨折情况：□无 □有 骨折次数：_____ 骨折部位_____  手术史：□无 □有 手术部位：________________  药物过敏史：_________________________________  最近服用药物：­_______________________________ | | |
| 现病史： □大骨节病 □骨关节炎 □风湿 □类风湿 □骨质疏松 □糖尿病 □肾炎 □高血压 □肿瘤 □心脏病 □其他 | | | | | |
| 关节功能障碍评分：   1. 关节休息痛：□无 □疼痛但不影响睡眠 □疼痛难忍应先睡眠需服止痛药 2. 关节运动痛：□无 □上下坡（楼梯）或行走15分钟以上路程有疼痛 □上下（楼梯）或行走少于15分钟以上路程有疼痛明显不能坚持 3. 晨僵： □无 □晨起关节屈伸僵硬时间少于15分钟 □晨起关节屈伸僵硬时间15分钟以上 4. 最大步行距离：□正常 □步行1公里以上有困难 □步行少于1公里有困难 5. 四肢活动能力：□正常 □立正下蹲或伸肘时有疼痛，但能达到正常活动1/2以上 □立正下蹲或伸肘时有疼痛，但不能达到正常活动1/2 | | | | | |
| 调查时间： | | | | | |

**Adult Questionnaire**

| Province_______________ | City ________________ | County ________________ | | Township (town) _________ | Village _________________ |
| --- | --- | --- | --- | --- | --- |
| Name: | Gender：□Male □Female | Age： | | Nationality： | Survey Number： |
| Living habits  Drinking：□Never □Occasional □Regular □Daily  Smoking：□Yes □No  Main food：□Rice □Rorn □Flour  Years of residence in the area：____________years | | | Anamnesis：__________________________________________________  _____________________________________________________________  Fracture condition：□No □Yes  Number of fractures：_____ Fracture site _____  Surgery history：□No □Yes Operative site：_________________  History of Drug Allergy：__________________________________  Drugs recently taken：­____________________________________ | | |
| Current diseases： □Kashin-Beck disease □Osteoarthritis □Rheumatism □Rheumatoid arthritis □Osteoporosis □Diabetes □Nephritis □Hypertension □Cancer □Heart disease □Others | | | | | |
| Joint dysfunction score：   1. Joint rest pain：□No □Pain but does not affect sleep □Pain unbearable, sleep need to take pain medication 2. Joint movement pain：□No □Up and down (staircase) or walking for 15 minutes or more feel pain □Up and down (stairs) or walking less than 15 minutes feel pain and can not go on 3. Morning stiffness：□No □Morning joint flexion and extension stiffness time was less than 15 minutes □Morning joint flexion and extension stiffness time was more than 15 minutes 4. Maximum walking distance：□Normal □Difficult to walk more than 1 kilometers □Difficult to walk less than 1 kilometers 5. Limb activity ability：□Normal □Pained when squatting or elbowing, but can achieve more than the normal activities of 1/2 □Pained when squatting or elbowing, but can not achieve more than the normal activities of 1/2 | | | | | |
| Date： | | | | | |
