# Supplementary material for: Prevalence of hand osteoarthritis and knee osteoarthritis in Kashin-Beck disease endemic areas and non Kashin-Beck disease endemic areas: A status survey
Source: PLoS One. 2018 Jan 10;13(1):e0190505. doi: 10.1371/journal.pone.0190505 (PMC5761882; doi:10.1371/journal.pone.0190505)
Supplement: S1 File — (DOCX) [file pone.0190505.s002.docx]

研究简介和自愿参加本项目研究的知情同意书

本研究目的在于寻找可以用来评估成人大骨节病治疗效果的生物指标，为成人大骨节病的治疗提供客观标准。按照《2011年度地方病防治项目实施方案》的内容和要求，在大骨节重病区，为贫困的大骨节患者提供就职服务，缓解患者临床症状，改善生活质量，主要为II度和III度患者免费提供能缓解症状、改善关节功能的治疗药物（国家准字号药物），但由于药物不是适合每个患者，所以需要通过问卷向您了解一些情况，并为你做健康检查，此外还将需要在治疗前后采取你的尿样和血样，采样过程，安全可靠。您所提供的所有个人信息我们将严格保密，您的姓名也不会在任何研究报告中。对收集的血样和尿样，我们将检测其中的相关指标，用来判定药物的治疗效果。所有研究结果只作为科学研究用，为成人大骨节的治疗提供客观依据。如您愿意参加，请在下表签名。即使签名加入研究后，也可随时退出。对您的积极配合我们表示衷心的感谢！

我已了解这项研究的目的、性质、和具体内容，并自愿参加。

参加者签名：_______________________日期：_________年_______月________日

**Introduction to research and informed consent**

The purpose of this study is to find biomarkers that can be used to assess the efficacy of adult Kaschin-Beck disease treatment and provide objective criteria for the treatment. In accordance with the content and requirements of the "2011 annual endemic disease prevention and control project implementation plan", in the Kaschin-Beck disease area, we provide services for the poor in Kaschin-Beck patients to improve the quality of life, mainly free to provide drugs (national quasi-word drugs) to the II and III degree of patients to help alleviate the symptoms and improve the functions of the joint treatment, but because the drugs are not suitable for all patients, so you need to pass through the questionnaire to understand some of the situation and we will do health checks for you, in addition to we need to take your urine and blood samples(sampling process is safe and reliable) before and after treatment. All of the personal information you provide will be kept strictly confidential and your name will not appear in any research report. For the collection of blood samples and urine samples, we will test the relevant indicators to determine the therapeutic effect of drugs. All the results of the study only as a scientific research for the treatment of adult Kaschin-Beck disease to provide an objective basis. If you would like to attend, please sign in the form below. Even if the signature is added to the study, you can leave at any time. Thank you for your active cooperation!

I have understood the purpose, nature, and specific content of this study and volunteered to participate.

Name：_______________________ Date：_____________________
